# Annotations of four high-quality indigenous chicken genomes identify more than one thousand missing genes in subtelomeric regions and micro-chromosomes with high G/C contents

**DOI:** 10.1186/s12864-024-10316-z

**Published:** 2024-05-01

**Authors:** Siwen Wu, Tengfei Dou, Sisi Yuan, Shixiong Yan, Zhiqiang Xu, Yong Liu, Zonghui Jian, Jingying Zhao, Rouhan Zhao, Xiannian Zi, Dahai Gu, Lixian Liu, Qihua Li, Dong-Dong Wu, Junjing Jia, Changrong Ge, Zhengchang Su, Kun Wang

**Affiliations:** 1 Present Address: Faculty of Animal Science and Technology, Yunnan Agricultural University, Kunming, Yunnan China; 2 Present Address: Department of Bioinformatics and Genomics, the University of North Carolina at Charlotte, Charlotte, NC 28223 USA; 3 State Key Laboratory of Genetic Resources and Evolution, Kunming Institute of Zoology, Chinese Academy of Sciences, Kunming, China; 4 Center for Excellence in Animal Evolution and Genetics, Chinese Academy of Sciences, Kunming, China

**Keywords:** Chicken, Gene annotation, New genes, RT-qPCR, Missing genes, Domestication, Evolution

## Abstract

**Background:**

Although multiple chicken genomes have been assembled and annotated, the numbers of protein-coding genes in chicken genomes and their variation among breeds are still uncertain due to the low quality of these genome assemblies and limited resources used in their gene annotations. To fill these gaps, we recently assembled genomes of four indigenous chicken breeds with distinct traits at chromosome-level. In this study, we annotated genes in each of these assembled genomes using a combination of RNA-seq- and homology-based approaches.

**Results:**

We identified varying numbers (17,497–17,718) of protein-coding genes in the four indigenous chicken genomes, while recovering 51 of the 274 “missing” genes in birds in general, and 36 of the 174 “missing” genes in chickens in particular. Intriguingly, based on deeply sequenced RNA-seq data collected in multiple tissues in the four breeds, we found 571 ~ 627 protein-coding genes in each genome, which were missing in the annotations of the reference chicken genomes (GRCg6a and GRCg7b/w). After removing redundancy, we ended up with a total of 1,420 newly annotated genes (NAGs). The NAGs tend to be found in subtelomeric regions of macro-chromosomes (chr1 to chr5, plus chrZ) and middle chromosomes (chr6 to chr13, plus chrW), as well as in micro-chromosomes (chr14 to chr39) and unplaced contigs, where G/C contents are high. Moreover, the NAGs have elevated quadruplexes G frequencies, while both G/C contents and quadruplexes G frequencies in their surrounding regions are also high. The NAGs showed tissue-specific expression, and we were able to verify 39 (92.9%) of 42 randomly selected ones in various tissues of the four chicken breeds using RT-qPCR experiments. Most of the NAGs were also encoded in the reference chicken genomes, thus, these genomes might harbor more genes than previously thought.

**Conclusion:**

The NAGs are widely distributed in wild, indigenous and commercial chickens, and they might play critical roles in chicken physiology. Counting these new genes, chicken genomes harbor more genes than originally thought.

**Supplementary Information:**

The online version contains supplementary material available at 10.1186/s12864-024-10316-z.

## Background

Chicken (*Gallus gallus*) provides us with most protein sources in our daily life and also is a model organism to study the development, immunity and diseases of vertebrates [1]. As a result, multiple versions of chicken genomes have been assembled, such as those for the red jungle fowl (galgal2-galgal5 and GRCg6a) [2–4], the broiler (GRCg7b) and the layer (GRCg7w) [5, 6]. However, the understanding of chicken genetics, egg and meat production, domestication and evolution is still limited due to the incomplete assemblies and annotations of these genome versions. Moreover, the unavailability of high-quality genome assemblies of diverse indigenous chickens and their annotations further worsens the issue. For example, the number of protein-coding genes encoded in chicken genome is still an issue of debate. On the one hand, like other birds, chickens have a small genome that is about a third of the sizes of other tetrapods’ genomes, resulted from large-scale segmental deletions in the avian lineage during evolution [2]. The reduction of genomes leads to a large number of gene loss, thus chickens and birds in general might have fewer genes than other tetrapods [7]. On the other hand, dozens to hundreds of genes that are essential in other tetrapods are believed simply missing in chickens and other birds’ genomes due to their incomplete assemblies, in particular, the micro-chromosomes where both gene density and G/C contents are higher [8, 9]. More recently, Li et al. [10] assembled a chicken pan-genome based on genomic data from 20 diverse breeds and identified a total 1,335 new genes. However, more than half of these new genes are micro-open reading frames (ORFs) with a coding DNA sequence (CDS) shorter than 300 bp, casting doubts on the authenticity of these “new genes”.

We recently sequenced and assembled genomes of four indigenous chicken breeds with unique morphological traits from Yunnan province, China, including Daweishan, Hu, Piao and Wuding chicken, using a combination of long reads, short reads, and Hi-C reads [11]. These chromosome-level assemblies are of higher or comparable quality with the recently released chicken reference genomes GRCg7b/w [11], providing us an opportunity to survey the repertoire of genes, particularly, protein-coding genes encoded in genomes of diverse chicken breeds. In this study, we annotated genes in each of these assembled indigenous chicken genomes using a pipeline that combines homology-based and RNA-seq-based methods. In addition to most protein-coding-genes annotated in the reference genomes (GRCg6a, GRCg7b/w), we identified a total of 1,420 new protein-coding genes in at least one of the four indigenous chicken genomes but were missed in the annotations of the reference genomes. These newly annotated genes (NAGs) are much longer than previously reported new genes [10], and the two sets only have limited overlaps. Most of the randomly selected NAGs can be verified by RT-qPCR experiment, thus these NAGs are likely authentic. Most of the NAG also are encoded in at least one of the reference genomes. Counting these NAGs, chickens have a similar number of protein-coding genes as other tetrapods do.

## Methods

### Materials

The GRCg6a, GRCg7b and GRCg7w genomes and annotation files were downloaded from the NCBI Genbank with accession numbers GCF_000002315.6, GCF_016699485.2 and GCF_016700215.2, respectively. The GRCg6a, GRCg7b and GRCg7w assemblies represent the reference chicken genome assemblies of a RJF, a broiler and a layer, respectively. Our previously assembled four indigenous chicken genomes were downloaded from the NCBI Genbank with accession numbers GCA_030914265.2, GCA_030849555.2, GCA_030914275.2 and GCA_030979905.2. All the Illumina short DNA sequencing reads, RNA-seq reads of different tissues of the four indigenous chickens were downloaded from the NCBI SRA database with the accession number SRP487534.

### Real-time quantitative PCR (RT-qPCR) analysis

Three female individuals of Daweishan chicken aged 10 months, three female individuals of Hu chicken aged 7 months, three female individuals of Piao chicken aged 10 months and three female individuals of Wuding chicken aged 10 months were collected from corresponding chicken breed populations from the Experimental Breeding Chicken Farm of the Yunnan Agricultural University (Yunnan, China). Chickens were killed by electric shock to unconsciousness followed by neck artery bleeding. One to two grams of relevant tissues were aseptically collected from each individual chicken in a centrifuge tube within 20 min after sacrifice and immediately frozen in liquid nitrogen, then stored at -80℃ until use. Total RNA from each tissue sample was extracted using TRlzol reagents (TIANGEN Biotech, Beijing China) according to the manufacturer’s instructions. RT-qPCR was performed using the Bio-Rad CFX96 real-time PCR platform (Bio-Rad Laboratories. lnc, America) and SYBR Green master mix (iQTM SYBRGreen ® Supermix, Dalian TaKaRa Biotechnology Co. Ltd. Add). We randomly selected 42 putative new genes for RT-qPCR validations, and the primers and their annealing temperatures used for each gene are listed in Supplementary Note. The β-actin gene was used as a reference. Primers were commercially synthesized (Shanghai Shenggong Biochemistry Company P.R.C). Each PCR reaction was performed in 25 μl volumes containing 12.5 μl of iQ™ SYBR Green Supermix, 0.5 μl (10 mM) of each primer, and 1 μl of cDNA. Amplification and detection of products were performed with the following cycle profile: one cycle of 95 °C for 2 min, and 40 cycles of 95 °C for 15 s, annealing temperature (see Supplementary Note for each gene) for 30 s, and 72 °C for 30 s, followed by a final cycle of 72 °C for 10 min. The specificity of the amplification product was verified by electrophoresis on a 0.8% agarose gel and DNA sequencing. The 2^−ΔCt^ method was used to analyze mRNA abundance. All tissues in a chicken breed were analyzed with three biological replicates (three individual chickens) and each biological replicate with five technical replicates. The means and standard deviation of all these measurements were presented in the relevant figures.

### Protein-coding gene annotation

To annotate the protein-coding genes in the assembled indigenous chicken genomes, we masked the repeats in each genome using WindowMasker (2.11.0) with default settings [12], and then we annotated the protein-coding genes using a combination of homology-based and RNA-based methods. For homology-based annotation, we collected all the protein-coding genes, pseudogenes and their corresponding CDS isoforms or exons in GRCg6a, GRCg7b and GRCg7w as the templates. The protein-coding genes in these three assemblies were recently predicted by the NCBI eukaryotic genome annotation pipeline that uses a combination of mRNA- and protein-based homology methods and ab initio methods. We mapped all the CDS isoforms of protein-coding genes and exons of pseudogenes in GRCg6a, GRCg7b and GRCg7w to each of the assembled indigenous chicken genomes using Splign (2.0.0) with default settings [13]. For each template gene whose CDSs can be mapped to an assembled genome, we concatenate all the mapped CDSs to form a putative full-length CDS, and predict it to be an intact gene, if and only if its length is an integer time of three and the last three nucleotides form a stop codon but no stop codon appeared in the middle of the sequence. If the CDSs of a template gene can be mapped to multiple loci in an assembled genome, we consider the locus with the highest mapping identity. If the putative full-length CDS contains a premature stop codon in its middle (nonsense mutation), or its length is not an integer time of three (ORF shift mutation), we verify the mutations by mapping the DNA short reads from the same individual to the genomic locus using bowtie (2.4.1) [14] with no gaps and mismatches permitted. If the locus can be completely covered by at least 10 short reads at each nucleotide position, we consider the loss of function mutation (nonsense mutation or ORF shift mutation) is fully supported by the short reads, and predict the sequence to be a pseudogene. Otherwise, we consider the loss of function mutation is not supported by the short reads, and call the sequence a partially supported gene, because the loss of function mutation might be artificially caused by errors of the long reads that could not be corrected by our assembly pipeline. For a few selenoprotein template genes where an "opal" (UGA) stop-codon may encode a selenocysteine, we manually checked the mapped loci, and annotated a stop-codon UGA in the indigenous chicken genomes as a selenocysteine codon if its corresponding UGA codon in the template gene is so annotated.

For RNA-based annotation, we first mapped all of the RNA-seq reads from various tissues as well as from the mixture of tissues of the four chicken breeds to the rRNA database SILVA_138 [15] and filtered out the mapped reads. We then aligned the unmapped reads to each of the four indigenous chicken genome assemblies using STAR (2.7.0c) [16] with default settings. Based on the mapping results, we assembled transcripts in each chicken using Trinity (2.8.5) [17] with its genome-guided option. Next, we mapped the assembled transcripts in each chicken to its assembled genomes using Splign (2.0.0) [13] with default settings, and removed those that at least partially overlapped non-coding RNA genes (see below), protein-coding genes or pseudogenes predicted by the homology-based method. For each of the remaining assembled transcripts, if it contained at least one ORF, and the longest one was at least 300 bp, we predicted the locus corresponding to the longest one to be a protein-coding gene.

Additionally, to detect protein-coding genes that might be not included in the four assemblies but expression in the tissues, we de novo assembled transcripts using Trinity (2.8.5) [17] with its de novo option using RNA-seq reads that could not be mapped to any of the four assembled genomes.

### RNA-coding gene annotation

We annotated tRNA, rRNA, miRNA, snoRNA, telomerase RNA and SRP RNA using infernal (1.1.2) [18] with Rfam (v.14) database [19] as the reference. In addition, we predicted an assembled transcript longer than 1,000 bp but lacking an ORF as a lncRNA.

### Alternative isoforms detection

We used STAR (2.7.0c) [16] and Cufflinks (2.2.1) [20] with default settings to detect the alternative isoforms of the genes in each chicken breed.

## Results

### More than one thousand protein-coding genes missing in reference annotations are found in four indigenous chicken genomes

**Fig. 1:**
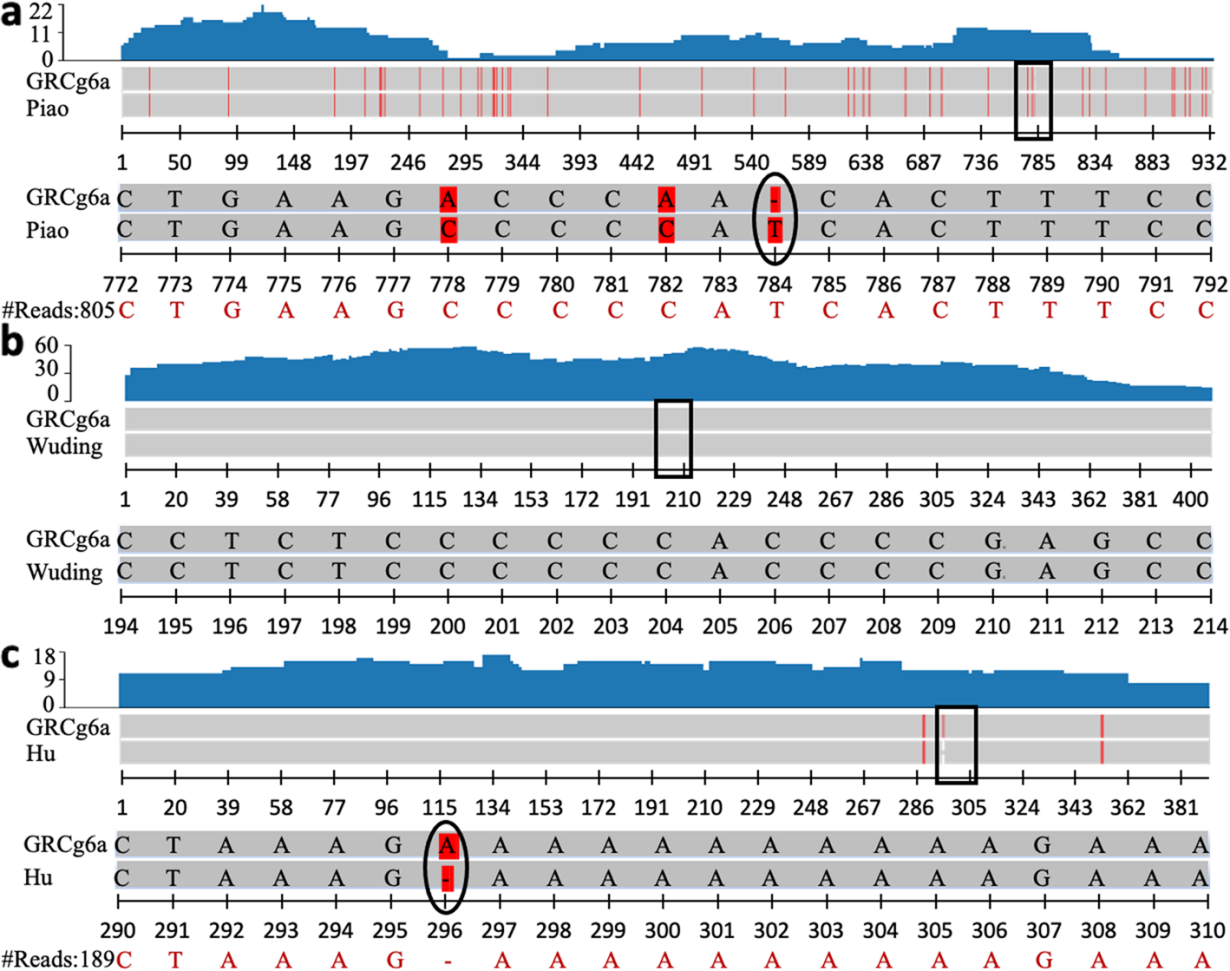
Examples of transcribed new genes and pseudogenes in the indigenous chickens and RJF. **a** A protein-coding gene in Piao chicken is predicted based on a pseudogene LOC107049240 in RJF that harbors a point deletion of ‘T’ at position 784, leading to an ORF shift. **b** A new gene predicted in Wuding chicken was also encoded in RJF. **c** A new gene predicted in Hu chicken is pseudogenized in RJF, due to an insertion of ‘A’ at position 296, leading to an ORF shift. The top blue graph indicates the RNA-seq expression level of the corresponding gene/pseudogene

By using a combination of homology-based and RNA-seq-based methods, we predicted varying numbers (17,497 ~ 17,718) of protein-coding genes in each of our recently assembled four indigenous chicken genomes [11] (Table 1), indicating the highly polymorphic nature of gene composition in various chicken breeds. Interestingly, these numbers of annotated genes in the indigenous genomes are similar to those annotated in the RJF genome GRCg6a (17,485), but fewer than those annotated in the broiler GRCg7b (18,024) and the layer GRCg7w (18,016) genomes. Specifically, we predicted 16,917 ~ 17,141 genes in each indigenous chicken genome based on homology to genes and pseudogenes annotated in GRCg6a, GRCg7b and GRCg7w (Methods). Of these homology-supported genes in each genome, 16,270 ~ 16,668 have an intact ORF (intact genes) (Table 1), and 473 ~ 647 contain either a premature nonsense mutation or an ORF shift mutation, which cannot be fully supported by short DNA reads from the chicken, however. It is highly likely that such “mutations” might be due to sequencing errors in long reads used to assemble the genomes that cannot be corrected by the short DNA reads. We therefore refer these genes as partially supported genes (Table 1). The vast majority of intact genes (98.7% ~ 98.8%) and partially supported genes (97.9% ~ 99.2%) in each genome are transcribed in at least one of the tissues that we examined using RNA-seq (Tables S1 ~ S4), suggesting that at least most of the homology-supported genes are likely authentic. Moreover, we predicted 6 ~ 7 genes in each indigenous chicken genome based on homology to pseudogenes in GRCg6a and/or GRCg7b/w (Table 1). These genes have an intact ORF that is fully supported by short DNA reads, and 4 ~ 7 are transcribed in at least one of the tissues examined (Tables S1 ~ S4), thus, they are likely to be functional. For example, the RJF pseudogene *LOC*107049240 at locus chr33:1,757,489 ~ 1,758,423 with a point deletion is mapped to chr33:2,240,289 ~ 2,241,224 of Piao chicken, encoding an intact ORF that is supported by 805 short DNA reads as well as large numbers of RNA-seq reads in multiple tissues (Fig. 1a, Table S3). Notably, the numbers of homology-supported genes in the indigenous chickens (16,917 ~ 17,141) are smaller than those annotated in GRCg6a (17,485), GRCg7b (18,024) and GRCg7w (18,016). We found that this was because 486 ~ 622 genes annotated in GRCg6a and GRCg7b/w became pseudogenes in each of the four indigenous chickens (Table 1). Moreover, we predicted 83 ~ 94 pseudogenes in the indigenous chickens based on homology to pseudogenes annotated in GRCg6a, GRCg7b and GRCg7w (Table 1).

**Table 1:**
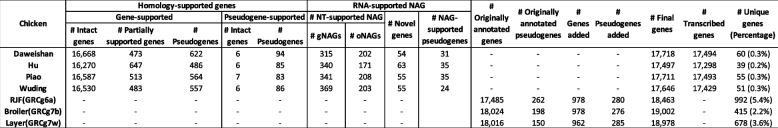
Summary of annotated protein-coding genes in the four indigenous chicken genomes in comparison with those in GRCg6a and GRCg7b/w

Based on RNA-seq data that could be mapped to the four assembled indigenous chicken genomes, we identified 12,543 ~ 13,930 putative genes in each of them (Table S5). Of these putative genes, 9,561 ~ 10,596 at least partially overlapped the homology-supported genes (Table S5), suggesting that homology-based method had already covered them. To avoid repeat, we did not consider them further. Of the remaining 2,982 ~ 3,334 genes that did not overlap homology-supported genes, 571 ~ 627 contained at least an ORF, and we considered them as NAGs (newly annotated genes), as they were not annotated in GRCg6a, GRCg7b or GRCg7w (Table 1). Since some of these NAGs in the four assembled genomes were highly similar (identity > 98.5%) to one another, we removed the redundancy and ended up with a total of 1,420 unique NAGs in the four genomes. Interestingly, 24 ~ 35 of the NAGs found in an indigenous chicken contained a premature nonsense mutation or an ORF-shift mutation, and thus became pseudogenes in at least one of the other three chickens (Tables 1 and S6). Of the 1,420 NAGs, 1,277 (89.9%) (511 ~ 572 in each of the four assembled genomes) are homologous to genes in the NT database (Table 1), so we refer them to as NT-supported NAGs. The remaining 143 (10.1%) (54 ~ 63 in each assembled genome) NAGs do not have a known homolog in NT database, and thus, we consider them to be novel genes (Table 1). NT-supported NAGs in each breed (511 ~ 572) are mapped to genes either in other breeds of *Gallus gallus* (315 ~ 369 or 60.9% ~ 66.5%) other than GRCg6a, GRCg7b and GRCg7w, or in other species (mainly avian species, 169 ~ 208 or 33.1% ~ 39.7%) (Table S7), suggesting that they are likely true genes. Among the 1,277 NT-supported NAGs, 793 are mapped to genes in other chicken breeds, and we refer them to as *Gallus gallus*-supported NAGs (gNAGs). For the remaining 484 NT-supported NAGs that are mapped to species other than *Gallus gallus,* we refer them to as other species-supported NAGs (oNAGs). Combining the 484 oNAGs with the 143 novel genes, we identified a total of 627 new genes that have not been reported in chickens (Table S6).

### The NAGs are widely encoded in chicken genomes and other genomes

**Fig. 2:**
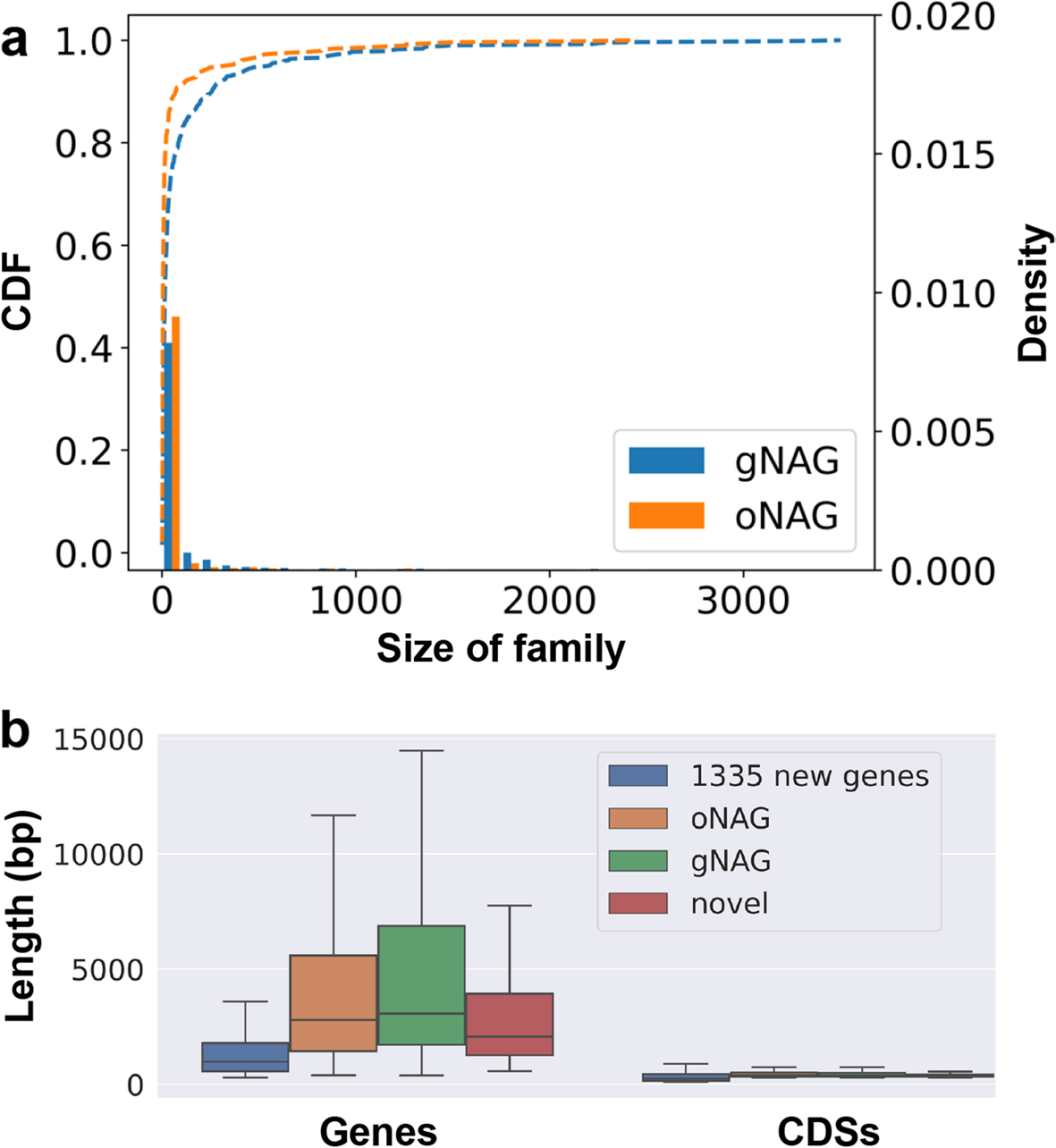
Distributions of family sizes and lengths of the NAGs. **a** Cumulative probabilities and densities of family sizes of the gNAGs and oNAGs. The dashed lines are the cumulative distribution function (CDF) of family sizes of the gNAGs and oNAGs. The histograms are the densities of family sizes of the gNAGsand oNAGs. **b** Comparison of the lengths of the NAGs and their CDSs with those of the previously predicted 1,335 new genes in 20 chickens

To see whether the NAGs also exist in the previously assembled chicken reference genomes but were simply missed by previous annotations, we mapped each of the 1,420 NAGs to GRCg6a, GRCg7b and GRCg7w assemblies. To our surprise, 1,291 (90.9%) of the 1,420 NAGs could be mapped to at least one of the GRCg6a (1,258), GRCg7b (1,254) and GRCg7w (1,247) assemblies, including 760 gNAGs, 410 oNAGs and 121 novel genes. Of the 1,291 mapped NAGs, 1,142 have intact orthologous ORFs in at least one of the GRCg6a (978), GRCg7b (978) or GRCg7w (962) assemblies (Table 1). For examples, a NAG in Wuding chicken at chr11:17,138,466 ~ 17,139,339 is mapped to RJF chr11:17,570,676 ~ 17,571,083 that encodes an intact ORF (Fig. 1b). Thus, most (1142, or 80.4%) of the 1,420 NAGs are encoded in these earlier assemblies but were missed by previous annotations (Table S6), due probably to the limited RNA-seq data used in annotation pipelines. Adding these intact NAG orthologs to the previously annotated lists of protein-coding genes, we increase the number of protein-coding genes in GRCg6a (18,463), GRCg7b (19,002) and GRCg7w (18,978) by 5.6% (978), 5.4% (978) and 5.3% (962), respectively (Table 1). Therefore, GRCg6a and GRCg7b/w encode more protein-coding genes than previously thought at the origin of the chicken genomics, supporting the previous conclusion inferred for birds in general [21]. On the other hand, 459 of the 1,291 mapped NAGs become pseudogenes in at least one of the GRCg6a (280), GRCg7b (276) and GRCg7w (285) assemblies (Table S6), i.e., they contain at least one premature nonsense mutation or one ORF shift mutation. For example, a NAG in Hu chicken at locus chr2:128,256,077 ~ 128,257,512 is mapped to RJF locus chr2:129,144,681 ~ 129,145,071 with an insertion of ‘A’ supported by 189 short reads, leading to an ORF shift (Fig. 1c). Thus, we also substantially increase the number of pseudogenes in GRCg6a (from 262 to 542), GRCg7b (from 198 to 474) and GRCg7w (from 150 to 435) (Table 1). Additionally, as we indicated earlier, the gNAGs also appear in other chicken breeds, while the oNAGs have homologs in other avian species. We thus estimated the family size of each of the gNAGs and oNAGs based on the number of hits that it had in the NT database. We found that both gNAGs and oNAGs had from two to thousands of family members, but in both cases, 90% of them had fewer than 300 members (Fig. 2a, Table S6). Moreover, the gNAGs tended to have larger family sizes than the oNAGs (*p* = 0.002, t-test) (Fig. 2a), indicating that the gNAGs are more widely distributed.

### The NAGs have limited overlaps with the previously identified 1,335 new genes in chicken genomes

We compared the 1,420 NAGs with the 1,335 new genes recently reported in 20 chicken genomes [10]. All the three groups of NAGs, i.e., gNAGs, oNAGs and novel genes, with a mean length of 8,232, 5,190 and 5,378 bp, respectively, are much longer than the 1,335 new genes with a mean length of only 1,413 bp (*p* = 8e-137, 7e-85 and 3e-22, respectively, Wilcoxon rank-sum test) (Fig. 2b). One of the reason for the discrepancy is that 756 (56.6%) of the 1,335 new genes are mini-ORFs [22] with a CDS length of 100 ~ 300 bp, while all of our 1,420 NAGs have a CDS length longer than 300 bp (Fig. 2b), suggesting that more than half of the earlier predicted so-called new genes might not be bona fide protein-coding genes. Of the 1,335 new genes, 660 (49.4%) can be mapped to at least one of the four indigenous chicken genomes with an identity greater than 98.5%. Of these 660 mapped new genes, 246 overlap our 214 predicted genes in one of the four chickens. Specifically, of these 246 so-called new genes, 196 overlapped the same number of our homology-supported genes in one of the four breeds and the remaining 50 overlapped 38 of our NAGs. Of the remaining 414 mapped new genes, 246 (59.4%) are actually mini-ORFs, and thus were not predicted as genes by our pipeline (Methods). Of the 1,335 new genes, 675 (51.6%) cannot be mapped to any of the four indigenous genomes. Of these unmapped so-called new genes, 510 (75.6%) are mini-ORFs, while the other 165 (24.4%) are longer than 300 bp that might be unique to the 20 chicken breeds used in the previous study [10]. Thus, though our 1,420 NAGs overlap 50 (3.7%) of the 1,335 so-called new genes, the two sets are quite different in terms of their lengths and overlapping rates.

### NAGs tend to have fewer alternative splicing isoforms than existing genes

Using the RNA-seq data collected in the tissues of each breed, we identified a total 24,660, 24,551, 24,927 and 25,281 alternative splicing isoforms of the homology-supported genes in the Daweishan, Hu, Piao and Wuding chicken genomes, respectively (Table 2). The NAGs expressed significantly fewer average numbers of isoforms than existing genes in all the breeds, except Hu chicken (Table 2). However, the novel genes expressed no significantly different average numbers of isoforms than the oNAGs and gNAGs, except Piao chicken (Table 2). These results suggest that the NAGs might be generally emerged more recently than existing genes and the novel genes might emerge at the same evolutionary periods as the oNAGs and gNAGs.

**Table 2:**
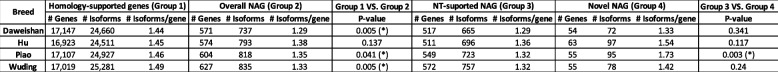
Comparison of detected isoforms in the four indigenous chicken breeds

Comparison between the indicated groups in each breed was done using two-tailed t-test

### The NAGs show strong tissue-specific expression patterns and can be experimentally validated

**Fig. 3:**
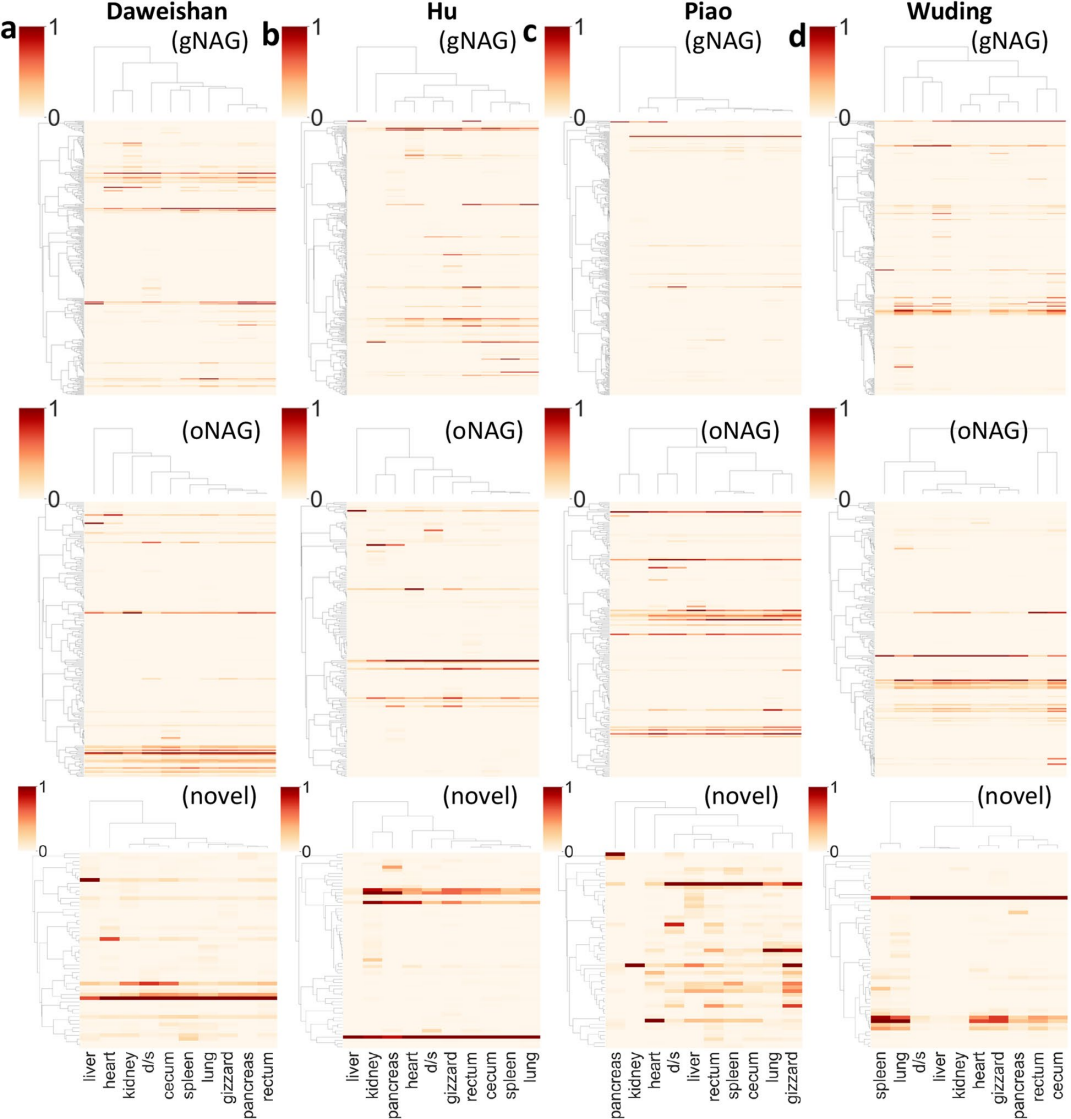
Expression levels of the NAGs in different tissues of the four indigenous chickens from RNA-seq data. **a** Expression levels of the NAGs in different tissues of the Daweishan chicken. **b** Expression levels of NAGs in different tissues of the Hu chicken. **c** Expression levels of the NAGs in different tissues of the Piao chicken. **d** Expression levels of the NAGs in different tissues of the Wuding chicken

**Fig. 4:**
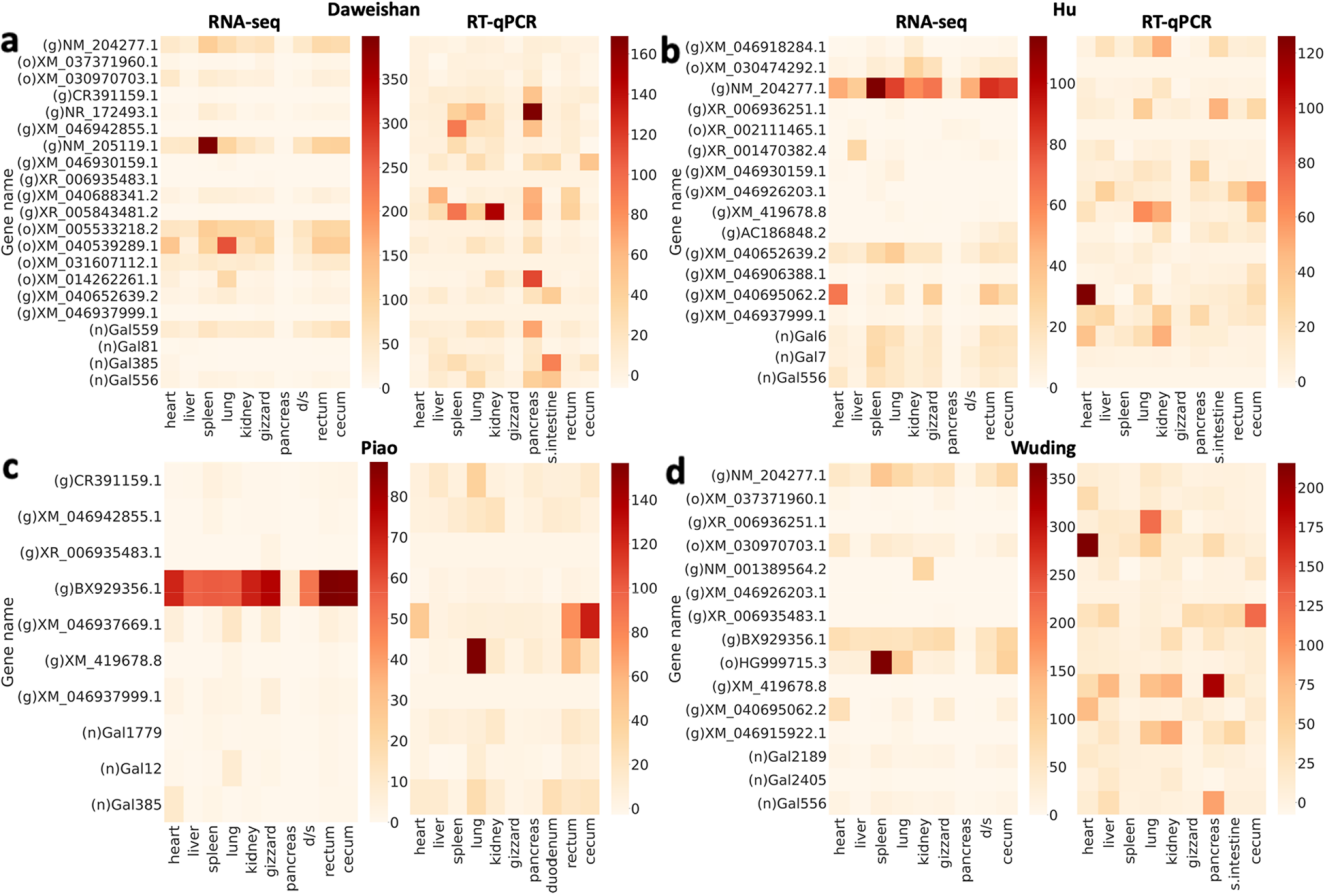
Heatmaps of expression levels of the 42 randomly selected NAGs in different tissues of the four indigenous chickens measured by RNA-seq and RT-qPCR. **a** Expression levels of NAGs in different tissues of the Daweishan chicken. **b** Expression levels of NAGs in different tissues of the Hu chicken. **c** Expression levels of NAGs in different tissues of the Piao chicken. **d** Expression levels of the NAGs in different tissues of the Wuding chicken. In each subfigure, genes with ‘g’ labels represent gNAGs, genes with ‘o’ labels represent oNAGs, and genes with ‘n’ labels represent novel genes. The NAGs that are not encoded in a genome are not shown in the breed. “d/s” represents duodenum/small intestine

As the first step to validate the NAGs, we examined their expression patterns using the RNA-seq data in ten tissues of each indigenous chicken (Methods). As shown in Fig. 3a ~ d and Tables S1 ~ S4, all the three groups of NAGs, i.e. gNAGs, oNAGs and novel genes, show strong tissue-specific expression patterns in all the four chicken breeds, suggesting that they are likely authentic genes as we argued earlier. To further validate the 1,420 NAGs, we randomly selected 42 of them and quantified their transcription levels in various tissues of the four chicken breeds using RT-qPCR. Of the 42 selected NAGs, eight are novel genes, nine are oNAGs, and the remaining 25 are gNAGs. Of the 42 NAGs, 21, 17, 10 and 15 were encoded in Daweishan, Hu, Piao and Wuding chickens, respectively (Tables S8 ~ S11), of which 18 (85.71%), 14 (82.35%), eight (80%) and 15 (100%) were expressed in multiple tissues of the four breeds, respectively (Fig. 4a ~ d). Combining the results from all the four breeds, 39 (92.86%) of the 42 NAGs were expressed in multiple tissues of at least one chicken breed. Of the 39 validated NAGs, 23 (59.0%), 6 (15.4%) and 10 (25.6%) were gNAGs, oNAGs and novel genes, respectively (Tables S8 ~ S11). Notably, the expression patterns in different tissues of the 39 NAGs measured by RT-qPCR are not very similar to those quantified by RNA-seq reads (Fig. 4a ~ d), which might be due to the different sensitivities of the two methods. Nonetheless, these results strengthen our conclusion that most of our identified NAGs are likely authentic.

### The NAGs tend to be located in the subtelomeric regions and micro-chromosomes with high G/C contents

**Fig. 5:**
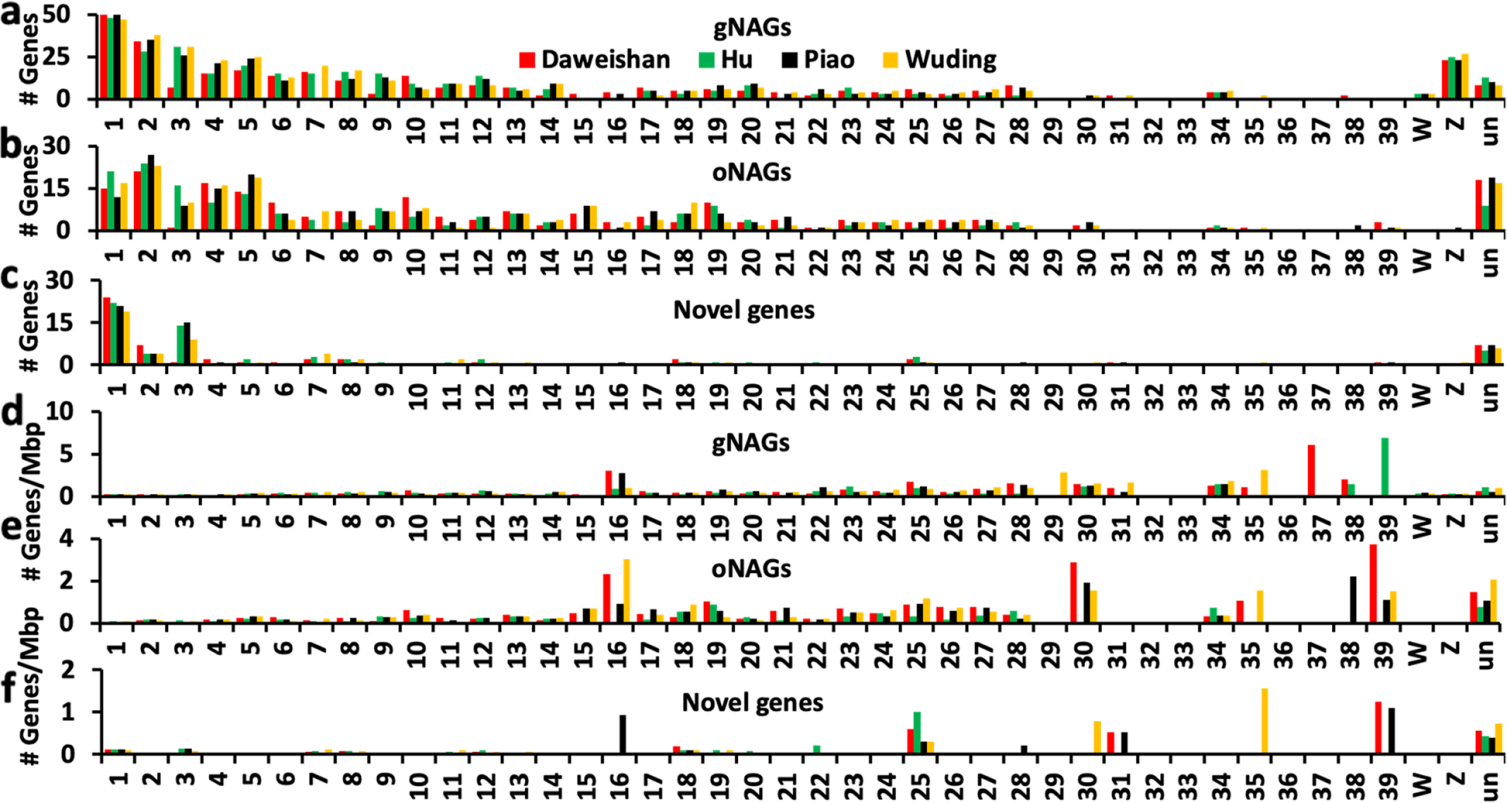
Distribution of different types of the NAGs found in the four indigenous chicken genomes. **a ~ c** Number of gNAGs, oNAGs and novel genes found on each chromosome and unplaced contigs. **d ~ f** Number of gNAGs, oNAGs and novel genes per million bp found on each chromosome and unplaced contigs

**Fig. 6:**
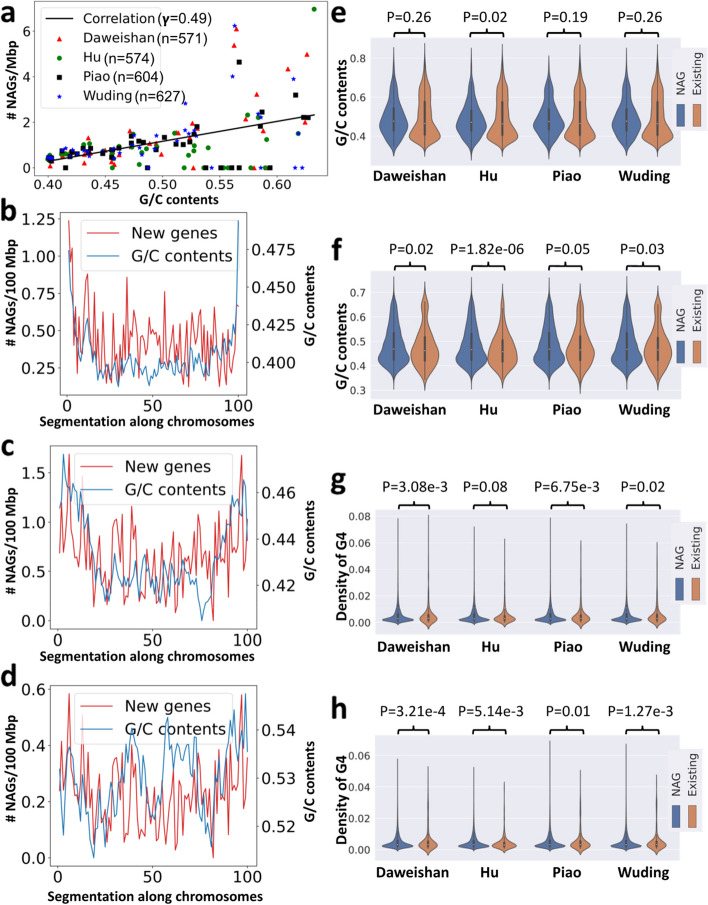
NAGs are preferentially found in genome regions with high G/C contents. **a** The relationship between the number of NAGs found in a 1-Mbp genome regions and its G/C contents in the four indigenous chicken genomes. The black line is the linear regression of data from the four chickens. **b** Average number of NAGs per million bp and average G/C contents along evenly divided 100 segments of the macro-chromosomes in the four chicken genomes. **c** Average number of NAGs per million bp and average G/C contents along evenly divided 100 segments of the middle chromosomes in the four chicken genomes. **d** Average number of NAGs per million bp and average G/C contents along evenly divided 100 segments of the micro-chromosomes in the four chicken genomes. **e** Comparison of G/C contents of the NAGs with those of existing genes. **f** Comparison of G/C contents of the 1-Mbp flanking regions of the NAGs with those of existing genes. **g** Comparison of G4 frequencies of the NAGs with those of existing genes. **h** Comparison of G4 frequencies of the 1-Mbp flanking regions of the NAGs with those of existing genes

We examined the distributions of the NAGs along the chromosomes. As shown in Fig. 5a ~ c, the NAGs are distributed on almost all the chromosomes, but have higher densities on micro-chromosomes and unplaced contigs (Fig. 5d ~ f). As micro-chromosomes have higher G/C contents than macro-chromosomes [23], we hypothesize that NAGs are preferentially found in chromosomes or chromosome regions with higher G/C contents. To test this, we segmented each chromosome in each genome into 1-Mbp regions, and computed the G/C contents in each segment and the number of NAGs found in it. As shown in Fig. 6a, the numbers of NAGs found in the regions are highly positively correlated with their G/C contents (Pearson correlation coefficient $\upgamma$=0.49). Thus, indeed, the higher G/C contents of a genome region, the larger number of NAGs are found in it. As G/C contents vary in different positions along chromosomes, we analyzed the relationships between the density of NAGs and their relative locations along the chromosomes. To this end, we grouped the chromosomes in three groups according to their sizes, i.e. macro-chromosomes (chr1-chr5 and chrZ), middle-chromosomes (chr6-chr13 and chrW) and micro-chromosomes (chr14-chr39). We binned each chromosome in each group in 100 equal portions and computed the G/C contents in each portion and the number of NAGs in it. Although both macro-chromosomes (Fig. 6b) and middle chromosomes (Fig. 6c) have fairly even G/C contents in their middle, with a mean value of 40% and 42%, respectively, G/C contents in their subtelomeric regions elevate substantially. The density of NAGs follows a similar pattern as the G/C contents along the bodies of the two groups of chromosomes (Fig. 6b and c). Thus, the NAGs are more likely found in the subtelomeric regions in macro-chromosomes and middle chromosomes. The elevation of G/C contents at the two ends of micro-chromosomes is less obvious (Fig. 6d), due probably to possible incomplete assembling of their telomeric regions and also to the high mean G/C contents (53%) along these chromosomes.

These results lead us to hypothesize that the high G/C contents and local structures, such as higher frequencies of quadruplexes G (G4) in NAGs and their surrounding regions, might prevent them and their transcripts from being sequenced. To test this, we compared the G/C contents and G4 frequencies in the NAGs and two 1-Mbp flanking regions at their two ends with those of existing genes in each genome. We found that NAGs had similar G/C contents to existing genes in all the four breeds except in Hu chicken (Fig. 6e). However, the flanking regions of NAGs had significantly higher G/C contents than those of existing genes in all the four breeds (Fig. 6f). Moreover, both NAGs and their flanking regions had significantly higher G4 frequencies than existing genes and their flanking regions, respectively, in all the four breeds (Fig. 6g and h). Thus, it is highly likely that the higher G4 frequencies in NAGs as well as higher G/C contents plus higher G4 frequencies in their surrounding regions render them and their transcripts more recalcitrant to sequencing technologies, and thus missed by the earlier chicken genome assemblies and annotations.

### The NAGs are involved in critical cellular functions

**Fig. 7:**
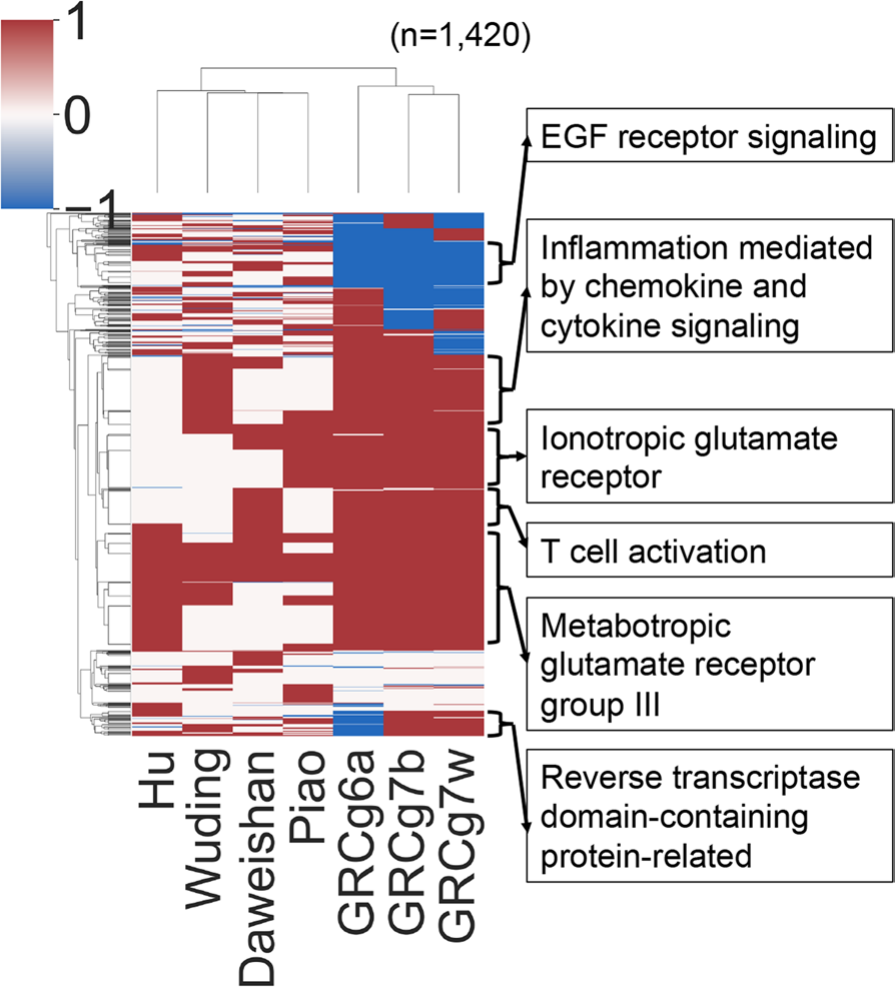
The heatmap of two-way hierarchical clustering of the 1,420 new genes based on their appearance (1, brown), absence (0, white) and pseudogenization (-1, blue) patterns in the seven chicken genomes

After demonstrating the wide distribution of the NAGs in chicken genomes, we asked whether there were any patterns for the NAGs to occur in these genomes. To this end, we clustered the chickens based on the similarity of presence, absence or loss of function mutation of the 1,420 NAGs in the genomes. As shown in Fig. 7, two distinct cluster can be seen, one is formed by the four indigenous chickens and the other is formed by GRCg6a, GRCg7b and GRCg7w. Specifically, the former cluster is featured by the fact that only 40.2 ~ 44.2% (571 ~ 627) of the NAGs occur while the remaining 55.8 ~ 59.8% (793 ~ 849) are either absent or become pseudogenes in the four indigenous chickens (Fig. 7, Table S6). In contrast, the latter cluster is distinct by the fact that two thirds (962 ~ 978) of the NAGs appear while the remaining one third (422 ~ 458) are either absent or become pseudogenes in GRCg6a, GRCg7b and GRCg7w. Moreover, we also clustered the NAGs based on their occurring patterns in the genomes and found that they are clustered in multiple distinct groups (Fig. 7). Although most of the NAGs do not have gene ontology (GO) [24] term assignments, those with GO terms are involved in a total of 40 GO biological pathways (Table S12), many of which correspond to gene clusters shown in Fig. 7. These GO biological pathways are involved in critical cellular functions, including transcription regulation, signal transactions, immunity, cell growth, metabolism and apoptosis, to name a few (Table S12), indicating that many of them might be critical for chicken biology.

### “Missing” protein-coding genes are found in chicken genomes

It has been reported that 274 genes that are widely encoded in reptiles and mammals are missing in avian species in general, and another 174 genes are missing in chicken (based on the galGal4 assembly) in particular [7]. However, some of these two sets of presumed missing genes have been recovered in chicken and other bird genomes [8, 9, 25]. We noted that 56 of the 274 missing genes in avian species and 43 of the 174 missing genes in chicken are annotated in at least one of the GRCg6a, GRCg7b or GRCg7w assemblies. To see whether our annotation recover any of the presumed missing genes, we mapped their human CDSs to each of the indigenous chicken genomes. We found that 48 and 36 of them, respectively, were among our predicted genes (Table S13). However, eight (ITPKC, TEP1, BRSK1, PLXNB3, MAPK3, HIGD1C, KMT5C and PACS1) of the 274 missing genes in avian species and seven (PPP1R12C, CCDC120, ATF6B, ASPDH, DDIT3, ADCK5, GPAA1) of the 174 missing genes in chicken, which were annotated in GRCg6a, GRCg7b or GRCg7w, did not appear in any of the four indigenous genomes (Table S13). It has been shown that RNA-seq data from multiple tissues can be used to recover presumed missing genes in birds [8, 9, 25]. To see whether more missing genes could be recovered by RNA-seq data collected in various tissues of the indigenous chickens, we de novo assembled transcripts using RNA-seq reads that could not be mapped to any of the four chicken genomes. Three of 34 such predicted CDSs/genes (Table S14) in the four chickens can be mapped to three (MAPK3, SLC25A23 and HSPB6) of the 274 missing genes in birds (Table S13A). MAPK3 is annotated in GRCg7b/w but missing in GRCg6a, while SLC25A23 and HSPB6 are missing in all these three previous annotations. Thus, loci of genes MAPK3, SLC25A23 and HSPB6 might be missed by the assemblies of the four indigenous chickens due probably to their refractory to the sequencing technologies used to assemble the genomes, and thus these genes are missed by our annotation pipeline.

### RNA genes in the chicken genomes

We also annotated RNA genes in each of the four indigenous chicken genomes. As summarized in Table S15, the four indigenous chicken genomes encode similar numbers of all the eight categories of RNA genes as annotated in GRCg6a and GRCg7b/w, except for snRNA, SRP (signal recognition particle) RNA and lncRNA. For snRNA, the four indigenous chicken genomes encode only three genes, while 66–75 were annotated in the GRCg6a and GRCg7b/w. For SRP RNA, the four indigenous chicken genomes encode 18 ~ 19 genes, while only one was annotated in GRCg6a and GRCg7b/w. For lncRNA, the four indigenous chicken genomes encode 13,429 ~ 15,080 genes, while only 8,233 ~ 10,062 were annotated in GRCg6a and GRCg7b/w.

### Mitochondrial genomes of the chickens

We also annotated genes in our previously assembled mitochondrial genomes of the four indigenous chickens. We found 13 protein-coding genes on the mitochondrial genomes of Hu, Piao and Wuding chickens, which are the same as annotated in GRCg6a and GRCg7b (as a paternal genome, GRCg7w does not contain the mitochondrion). Interestingly, in the Daweishan mitochondrial genome, we found that ND5 (CDSs length = 1,818 bp in RJF) is a pseudogene because of an ORF-shift mutation caused by a five-bp deletion at CDS positions 1,552 ~ 1,556, affecting 89 amino acids at the carboxyl terminus of the protein.

## Discussion

Although multiple improved versions of the RJF genome (galgal2-galgal5 and GRCg6a) [2–4] and commercial chicken genomes (GRCg7b/w) [5, 6] have been assembled, understanding of chicken genomes is still far from complete. In particular, the number of protein-coding genes annotated in genomes of different breeds varies widely, and many presumed missing genes in chickens are still not found [7]. To better understand the gene repertoire in various chicken breeds and the underlying reasons of missing genes, in this study, by using a combination of homology-based and RNA-seq-based methods, we annotated the protein-coding genes in four indigenous chicken genomes assembled at chromosome-level with high-quality. We annotated varying numbers (17,494 ~ 17,718) of genes in the four indigenous chicken genomes, which are similar to that previously annotated in the GRCg6a (17,485), but smaller than those previously annotated in GRCg7b (18,027) and GRCg7w (18,016). However, of the genes that we annotated in each of the indigenous chicken genomes, from 511 to 572 are not seen in the reference annotations of GRCg6a and GRCg7b/w. After removing the redundancy, we ended up with a total of 1,420 NAGs, of which 143 might be novel genes with no homologs in the NT database, 484 are homologs to genes in other specie (most of which are avian) (oNAGs), and 793 are mapped to known chicken genes in other chicken breeds other than GRCg6a and GRCg7b/w (gNAGs). The tissue-specific expression of these NAGs and RT-qPCR validations of randomly selected NAGs in multiple tissues of at least one breed suggest that they are likely authentic. Combing the oNAGs and novel genes, we identified 627 new chicken genes.

Interestingly, although the NAGs are found in almost all the chromosomes, they are more likely found in genomic regions with high G/C contents, such as micro-chromosomes and subtelomeric regions of macro-chromosomes and middle chromosomes. Although the 1,335 new genes recently reported by Li et al. in 20 chicken genomes also were preferentially found in subtelomeric regions [10], they had only limited overlaps with our NAGs, and two sets differed largely in their lengths. The discrepancy might be due to different numbers of chickens/breeds used and different definitions of a gene adopted in the two studies. For example, more than half (756, 56.6%) of the 1,335 previously identified genes have a CDS length of only 100 ~ 300 bp, thus they might be mini-ORFs [22] and not be bona fide protein-coding genes.

Moreover, we identified 48 of the 274 missing genes in birds in general, and 36 of another 174 missing genes in chicken in particular, in at least one of the four assembled indigenous genomes (Table S13). We also uncovered three of the 274 missing genes in birds (Table S13A) by mining unmapped RNA-seq reads. Two of the three recovered genes are missing in the GRCg6a and GRCg7b/w annotations. Our ability to identify the 1,420 NAGs and to recover missing genes indicates that the approach that we used to annotate the indigenous chicken genomes have largely overcome the limitations of the earlier methods. However, the assembled chr16 and some other micro-chromosomes of the four indigenous chickens are still not complete enough, thus, none of our 1,420 NAGs recalls any of the 274 missing genes in birds in general, and of additional 174 missing genes in chickens in particular. Therefore, if the remaining presumed missing genes are encoded in the chicken genomes, accurate-enough ultra-long-reads (> 100 kbp) from new sequencing technologies that allow the recent telomere to telomere assembly of a hypoploid human genome [26] might be needed to completely assemble the chicken genomes, thereby recovering the still missing genes.

Interestingly, 1,142 (80.4%) of the 1,420 NAGs also are encoded in at least one the GRCg6a, GRCg7b or GRCg7w assemblies (Table S6), but were missed by the earlier annotation pipelines. Our findings might suggest a reason for such missing. We showed that NAGs tend to have higher G4 frequencies, and their surrounding regions tend to have higher G/C contents and higher G4 frequencies than the corresponding regions of existing genes. It is well known that genome regions and transcripts with high G/C contents and G4 frequencies are more refractory to existing sequencing technologies [27]. Although the more recently released reference genomes include most of the NAGs, the annotation pipeline still missed them due probably to insufficient RNA-seq data used. Our deeply sequenced RNA-seq data collected from multiple tissues in the four chicken breeds clearly cover these genes, and thus help us to identify them.

The wide presence of the NAGs in these diverse chicken genomes indicates that most of the NAGs might play crucial roles in critical cellular functions as also indicated by GO pathways that they are involved in (Table S12). Including the 1,420 NAGs and recovered missing genes, we annotate 17,497 ~ 17,718 protein-coding genes in the four indigenous chicken genomes and increase the number of protein-coding genes in GRCg6a, GRCg7b and GRCg7w to 18,463, 19,002, 18,978, respectively. Considering that many gene-rich micro-chromosomes are still not fully assembled and that some chromosomes such as chr16 contain highly polymorphic regions [11], the number of annotated genes may increase further once all the micro-chromosomes are fully assembled and polymorphic regions are sufficiently explored. Thus, the chicken genomes might encode highly varying yet similar numbers of protein-coding genes as other tetrapods (18,000 ~ 25,000) [5] such as humans [28], as previously suggested for birds in general [21]. The highly varying numbers of protein-coding genes found in the seven chicken genomes that we analyzed in this study highlight the polymorphic nature of genes contents in various chicken breeds probably owing to their unique evolutionary and domestication histories. For example, the much larger number of protein-coding genes in the two commercial chickens (GRCg7b/w) than in RJF (GRCg6a) and indigenous chickens might be the results of the breeding programs used to produce the fast-growth and high egg-laying terminal commercial lines for production by taking advantage of hybrid vigor through a series of gene introgression from multiple purebred populations generated by intensive artificial selection [29–32].

Finally, we identified unexpectedly large numbers of pseudogenes in the four indigenous chicken genomes, since hundreds of genes in GRCg6a and GRCg7b/w and dozens of NAGs in other indigenous chickens became pseudogenes in each of them (Table 1). Moreover, as hundreds of NAGs became pseudogenes in GRCg6a and GRCg7b/w, we substantially increased the number of annotated pseudogenes in these three genomes. Thus, it appears that the RJF and commercial genomes harbor more pseudogenes than originally thought. However, to reveal the possible roles of the pseudogenes in chicken evolution and domestication, we need to conduct more detailed analyses of the evolutionary behaviors of the pseudogenes, such as their Ka/Ks ratios and fixation rates in relevant populations.

## Conclusions

In this study, we annotated assembled high-quality genomes of four indigenous chickens using a combination of homology- and RNA-seq based approaches. We not only recovered dozens of previously presumed “missing” genes in chickens, but also found a total of 1,420 NAGs that were missed by previous annotations. The NAGs are often found in subtelomeric regions of macro-chromosomes and middle chromosomes as well as in micro-chromosomes and some unplaced contigs, where G/C contents are high. Moreover, NAGs tend to have high G4 frequencies, and their surrounding regions tend to have both high G/C contents and high G4 frequencies. We verified 39 (92.9%) of 42 randomly selected NAGs using RT-qPCR experiments. The NAGs showed tissue-specific expression and are involved in many important biological pathways. Most of the NAGs also are encoded in the previous chicken genome assemblies. Counting the NAGs, chicken genomes encode more genes than originally thought.

### Supplementary Information

**Supplementary Material 1.**

**Supplementary Material 2.**

## Data Availability

The GRCg6a, GRCg7b and GRCg7w assemblies and annotation files are available from the NCBI Genbank at https://www.ncbi.nlm.nih.gov/datasets/genome/GCF_000002315.6/, https://www.ncbi.nlm.nih.gov/datasets/genome/GCF_016699485.2/ and https://www.ncbi.nlm.nih.gov/datasets/genome/GCF_016700215.2/, respectively. Our previously assembled four indigenous chicken genomes are available from the NCBI Genbank at https://www.ncbi.nlm.nih.gov/datasets/genome/GCA_030914265.2/, https://www.ncbi.nlm.nih.gov/datasets/genome/GCA_030849555.2/, https://www.ncbi.nlm.nih.gov/datasets/genome/GCA_030914275.2/ and https://www.ncbi.nlm.nih.gov/datasets/genome/GCA_030979905.2/. All the Illumina short DNA sequencing reads, RNA-seq reads of different tissues of the four indigenous chickens are available at https://www.ncbi.nlm.nih.gov/sra/?term=SRP487534. Annotation results of the four indigenous chickens and the three reference chickens (GRCg6a, GRCg7b and GRCg7w) are available from the FigShare at https://figshare.com/articles/dataset/Gene_annotation_results_of_four_indigenous_chickens/25608204.
